# Associations between Participation in a Ranger Program and Health and Wellbeing Outcomes among Aboriginal and Torres Strait Islander People in Central Australia: A Proof of Concept Study

**DOI:** 10.3390/ijerph15071478

**Published:** 2018-07-12

**Authors:** Roxanne Jones, Katherine A. Thurber, Alyson Wright, Jan Chapman, Peter Donohoe, Vanessa Davis, Raymond Lovett

**Affiliations:** 1 National Centre for Epidemiology and Population Health, Research School of Population Health, Australian National University, Acton ACT 2601, Australia; katherine.thurber@anu.edu.au (K.A.T.); alyson.wright@anu.edu.au (A.W.); jan.chapman@anu.edu.au (J.C.); raymond.lovett@anu.edu.au (R.L.); 2 The Central Land Council, 27 Stuart Highway, Alice Springs NT 0870, Australia; peter.donohoe@clc.org.au; 3 Tangentyere Research Hub, 4 Elder Street, Alice Springs NT 0871, Australia; vanessa.davis@tangentyere.org.au

**Keywords:** Ranger, culture, wellbeing, Indigenous, Aboriginal, Torres Strait Islander, land management

## Abstract

Culture can be viewed as an integral part of Aboriginal and Torres Strait Islander health and wellbeing. This study explores the association between caring for country, through participation in a Ranger program, and wellbeing. We analyzed cross-sectional data collected in Central Australia in 2017, comparing health and wellbeing (life satisfaction, general health, psychological wellbeing and family wellbeing) among Aboriginal and Torres Strait Islander peoples employed as Rangers (n = 43) versus not employed as Rangers (n = 160). We tested if any differences in outcomes were explained by differences in key demographic or health factors. Ranger participation was significantly associated with very high life satisfaction (PR = 1.69, 95% CI: 1.29, 2.20) and high family wellbeing (PR = 1.47, 95% CI: 1.13, 1.90); associations remained significant after individual adjustment for education, income, employment, health risk factors and health conditions. The magnitude and direction of associations were similar for very good general health, but results were not significant. We did not identify an association between Ranger participation and psychological wellbeing. While based on a small sample, these findings support the assertion that participation in the Ranger program is associated with positive health and wellbeing outcomes. This supports the continuation of cultural participation and practice through the Ranger program and has implications for funding, program and policy development.

## 1. Introduction

Aboriginal and Torres Strait Islander people are Australia’s First Peoples and their cultures are among the longest-continuing cultures in the world. For at least 65,000 years, Aboriginal and Torres Strait Islander people have developed and maintained cultural practices that are closely tied to their ancestral land [1]. Culture for Aboriginal and Torres Strait Islander people encompasses a wide range of beliefs, traditions and practices that have evolved over time; the expression of culture has also changed over time. Key cultural constructs for Aboriginal and Torres Strait Islander peoples include: connection to country; cultural beliefs and knowledge; language; family, kinship and community; expression and cultural continuity; and self-determination and leadership. Each of these constructs includes a range of sub-themes [2]. 

Culture is described as an integral part of Aboriginal and Torres Strait Islander health and wellbeing. This is aligned with holistic views of health, which perceive health as inclusive of the physical, social, emotional and cultural wellbeing of individuals, families and their communities [3,4]. While the existing evidence base is limited, a recent review of domestic and international qualitative and cross-sectional quantitative analyses supports a positive association between Indigenous cultures and health and wellbeing [2]. For example, increased involvement in caring for country activities was significantly associated with improved health outcomes (lower odds of diabetes, obesity, and psychological distress) and an improved health risk factor profile (more frequent physical activity, greater bush food consumption, lower systolic blood pressure, and lower cardiovascular disease risk [5]) in a sample of 298 Aboriginal and Torres Strait Islander residents in the Northern Territory of Australia [6]. Caring for country activities included spending time on country, burning of annual grasses, gathering food and medicinal resources, protecting sacred sites, and producing artwork [6,7,8].

The current study builds on the previous literature by exploring the association between Aboriginal and Torres Strait Islander wellbeing and caring for country, through participation in an Indigenous Ranger program. Ranger programs employ Aboriginal and Torres Strait Islander people who combine cultural knowledge and experience with land conservation to protect and manage the environment. Rangers typically engage in land management activities such as protection of sacred sites (culturally significant places) and endangered species, fire management, conservation of water bodies and invasive weed control. In undertaking land management activities, Rangers draw on customary cultural knowledge and practices of traditional owners and elders. In this paper, we consider participation in the Ranger program as a proxy for cultural engagement and caring for country.

Ranger programs have been established in all jurisdictions in Australia, primarily through the Australian Government’s Working on Country Program [9]. The Ranger programs are often facilitated by Land Councils and other Aboriginal and Torres Strait Islander led organizations. The programs have generated employment opportunities for Aboriginal and Torres Strait Islander people, and have improved biodiversity and land management outcomes [8,10,11,12]. There is also evidence indicating that participation in Ranger programs has economic benefits [13,14,15]. Given that participation in Ranger programs facilitates cultural engagement (for example, through caring for country and transfer of customary ecological knowledge and practices), the program may also have positive impacts on Aboriginal and Torres Strait Islander health and wellbeing. However, to date, we lack quantitative evidence on the health and wellbeing impacts of participation in Ranger programs.

To contribute to filling this gap, this study aimed to provide ‘proof of concept’ that participation in a Ranger program may have benefits for health and wellbeing, to form a foundation for large scale longitudinal research. To achieve this aim, this study compares the health and wellbeing of Aboriginal and Torres Strait Islander peoples employed as Rangers to those not employed as Rangers, and tests if differences in health and wellbeing among Rangers are independent of differences in education, employment, income, health conditions and health risk factors.

## 2. Methods

### 2.1. Research Approach

This study was conducted using a community-based participatory research approach. Community-based participatory research fosters partnerships between community and research agencies, with the aim of facilitating inclusivity and knowledge co-production [16]. This approach values the use of local knowledge for local action [17]. In contrast to research which has often been undertaken ‘on’ Aboriginal and Torres Strait Islander peoples rather than ‘with’ them, for the benefit of the researcher alone [18,19], community-based research redistributes power between researchers and those participating in the research. This research approach seeks to ensure reciprocity and restructure power relations, which are fundamental ethical principles for the conduct of research with Aboriginal and Torres Strait Islander peoples [20]. 

The current study was conducted in partnership with the Central Land Council (CLC) where a synergy was identified in late 2016. The CLC employs and supports Ranger groups to undertake appropriate care of their lands. The CLC were interested in evaluating the multiple benefits of their Ranger program, including wellbeing benefits. This aligned with the research team’s work in developing and testing a range of measures of cultural engagement, expression and practice with a diversity of Aboriginal and Torres Strait Islander groups across the country as part of a national longitudinal study. The CLC invited the research team to work with the Ranger group to develop and refine the cultural indicators, and to field test the survey with the Rangers. The researchers used the data collected through this field testing to provide evidence on the health and wellbeing of participants in the Ranger program, and also to further refine the survey for the study.

Preliminary results were presented to and discussed with the CLC and Rangers in 2017. Rangers’ interpretation of the results are incorporated in the discussion. Final results were presented to local organizations (including the CLC) and Rangers. Consent for publication was also obtained. A joint seminar to researchers and policy makers is planned for mid-2018. 

### 2.2. Setting

This study was conducted in Central Australia as part of the development phase of *Mayi Kuwayu: The national study of Aboriginal and Torres Strait Islander wellbeing* (the Mayi Kuwayu Study) and to assist the CLC in evaluating the potential benefits of their Ranger program. The Mayi Kuwayu Study will be a large-scale, national longitudinal study of adults (16 years and older) who identify as Aboriginal and/or Torres Strait Islander. The aim of the Mayi Kuwayu Study is to generate robust data to enable the quantification of cultural engagement, expression and practices, and its association with health and wellbeing. The Mayi Kuwayu Study has been developed in partnership with Aboriginal and Torres Strait Islander communities and organizations, including through 24 focus groups and field testing of the survey in 2 sites. These focus groups were critical to developing and refining measures of culture and wellbeing that are appropriate and meaningful to participants across the country. Details of the Mayi Kuwayu Study are provided elsewhere [21].

The current study is based on data collected through field testing of the Mayi Kuwayu Study survey with Aboriginal and Torres Strait Islander people in Central Australia between May and November 2017. This paper reports cross-sectional analysis of these data.

### 2.3. Recruitment and Study Population

Rangers working in Central Australia were invited to complete the survey while attending their annual professional development camp in May 2017. All Rangers at the camp were invited to participate in the survey and participation was voluntary. Participants were provided with a plain language information sheet and consent form. Participants could self-complete the consent form and survey, or complete this with an interviewer. Of approximately 80 Rangers present at the camp, 43 completed the survey. 

Non-Ranger participants were Aboriginal and/or Torres Strait Islander adults living in a similar geographic area to Rangers and have never been employed as a Ranger (referred to hereafter as non-Rangers). The recruitment and interviewing of non-Rangers was conducted by a local community organization. A purposive Indigenous field worker sampling approach was used for recruitment, with a local community researcher conducting the recruitment. In total, 160 non-Rangers completed the survey.

### 2.4. Data and Variables

All data used in this study are based on self-reported responses to the survey. Both self-completed and interviewer administered surveys are included in analysis.

#### 2.4.1. Ranger Status 

Participants were categorized as Rangers if they were involved in the Ranger program (full and part time), and as non-Rangers if they have not been employed as a Ranger.

#### 2.4.2. Outcome Variables 

Four health and wellbeing outcomes are included in this analysis: life satisfaction, general health, psychological wellbeing and family wellbeing. 

Life satisfaction was measured according to responses to the question, ‘How satisfied are you with your life as a whole’, on a scale from 0 (completely dissatisfied) to 10 (completely satisfied) [22]. Scores were categorized as low to high life satisfaction (score 0–8) or very high life satisfaction (score 9–10). 

General health was measured according to the question, ‘How would you rate your general health?’ [23]; response options were ‘poor, fair, good, very good or excellent’. Responses were categorized into two groups: poor to fair general health (poor or fair) and very good general health (good, very good or excellent). 

Psychological wellbeing was measured using the Kessler Psychological Distress (K5) scale, modified to include clarifying statements [24]. Responses to the five questions were summed; participants were categorized as having low/moderate (score 5–11) or high/very high levels of distress (score 12–25). Scores were only calculated for participants with complete data on the five items. For the analysis, those with ‘low/moderate distress’ were defined as having high psychological wellbeing; those with ‘high/very high distress’ were defined as having low/moderate psychological wellbeing.

Family wellbeing was measured using the Western Australian Aboriginal Child Health Survey family functioning scale [25], modified during the study development process. This was measured according to responses to a set of nine questions each with response options of ‘not at all’ (1) to ‘very much’ (5). Responses were summed (range: 9–45), and participants were categorized as having low/moderate (score 9–36) or high family wellbeing (score 37–45). Responses to the nine questions were summed for participants with complete data only; participants missing responses to any of the questions were coded as missing. 

#### 2.4.3. Sociodemographic Factors

Age was calculated based on date of birth and categorized as: 16–24, 25–34, 35–44, or >45 years. Highest attained qualification (education) was categorized into two groups: not completed Year 12 (no school, primary school and intermediate certificate); and, completed Year 12 or above (higher school, leaving certificate, diploma/certificate, trade or tertiary). Financial status was measured based on responses to the survey item, ‘Given your current needs and financial responsibilities, indicate if you are: very poor, poor, just getting along, reasonably comfortable, very comfortable or prosperous’. Responses were categorized as low financial status (very poor, poor or just getting along) or high financial status (reasonably comfortable, very comfortable or prosperous). Non-Rangers were categorized as employed if they reported working part or full-time or if they were studying; and categorized as not employed if they were not working (including being retired, on a pension or an unpaid carer). All Rangers were categorized as employed.

#### 2.4.4. Health Conditions and Health Risk Factors

Participants were asked if they had ever been told by a doctor that they had heart disease or diabetes. For analysis participants were coded as ever or never having each condition. We also created a composite health condition score that summed the number of conditions participants had ever reported (range: 0–2). Participants were categorized as having no (neither of the two health conditions) or any health conditions (one or more of the two health conditions). The health condition score was coded as missing if participants were missing data on either of the health conditions.

Participants were categorized as a current smoker or non-smoker (never or ex-smoker). Participants were asked if they had ever been told by a doctor that they had high blood pressure or high cholesterol; responses were categorized as ever or never for each. We also created a composite health risk score that summed the number of risks reported (range: 0–3). Participants were categorized as having no (none of the three health risk factors) or any health risk factor (one or more of the health risk factors). The health risk factor score was coded as missing if participants were missing data on any of the health risk factors.

### 2.5. Statistical Methods

#### 2.5.1. Descriptive Analysis

We conducted a descriptive analysis of the demographic factors, health conditions and risk factors, and wellbeing outcomes for the Ranger and non-Ranger samples separately. An established protocol was utilized to confidentialize small cells. Individual cells are suppressed where the cell contains 5 or fewer observations (n ≤ 5, with the exception of ‘missing’ category), such that it is not possible to identify the exact number in any category that has 5 or fewer observations. 

#### 2.5.2. Inferential Analysis

We used log-binominal models to calculate prevalence ratios (PRs) and 95% Confidence Intervals (CIs) for each outcome (life satisfaction, general health, psychological wellbeing and family wellbeing) for Rangers compared to non-Rangers. We opted to use log binomial regression models because our outcomes of interest were common [26]. All models excluded participants missing data on the outcome of interest (total included in models ranged from n = 162–178).

To test if differences in key demographic or health factors accounted for differences in wellbeing outcomes for Rangers compared to non-Rangers, we repeated each regression and individually adjusted for: education, employment status, financial status, health condition score, and health risk factor score. Given the small sample size, we did not have power to mutually adjust for all variables. Participants missing data on the exposure variable of interest were included as a separate missing category; as such, the total sample size was consistent for all models with the same outcome. Where a cell of the exposure variable contained missing data, we did not include a missing category in the regression (education and health risk score). Stata 14 was used for all analysis. 

### 2.6. Ethics

Ethics approval for Mayi Kuwayu and this study have been received from the following Human Research Ethics Committees (HRECs): The Australian Institute of Aboriginal and Torres Strait Islander Studies HREC (approval number: E030/22052015); the Aboriginal Health and Medical Research Council of New South Wales Ethics Committee (1268/17); Central Australian Human Research Ethics Committee (CA-17-2810); the Northern Territory Department of Health and Menzies HREC (2017–2804); the Australian National University HREC (2016/767); the University of Tasmania HREC (H0016473); Aboriginal Health Research Ethics Committee (14-07-723); St. Vincent’s Hospital Melbourne HREC (HREC 132/17); the Western Australian Aboriginal Health Ethics Committee (787); The Australian Government Department of Health (Project 10-2017). 

## 3. Results

### 3.1. Sample Characteristics

A total of 43 Rangers and 160 non-Rangers participated in the study; 60% (n = 26) and 31% (n = 50) of participants were male, respectively (Table 1). Participants ranged from 16 to 77 years in age, with a mean age of 37 years. All Rangers were employed whereas 33% (n = 52) of non-Rangers were employed. Forty-two percent (n = 18) of Rangers reported high financial status compared to 31% (n = 49) of non-Rangers. 

### 3.2. Health Conditions and Health Risk Factors 

Among Rangers, <11% (n ≤ 5) reported ever having heart disease and 28% (n = 12) diabetes, compared to 11% (n = 17) and 18% (n = 29) in the non-Ranger group, respectively (Table 2). Over a third (35%, n = 15) of Rangers had at least one of the two conditions, compared to only 21% (n = 34) of non-Rangers. Sixty-three percent (n = 27) of Rangers were current smokers, as were 56% (n = 89) of non-Rangers.

### 3.3. Wellbeing

Sixty percent (n = 26) of Rangers reported very high life satisfaction compared to 36% (n = 58) of non-Rangers. Forty-seven percent (n = 20) of Rangers reported very good general health compared to 38% (n = 61) of non-Rangers. Sixty-three percent (n = 27) of Rangers reported high psychological wellbeing compared to 53% (n = 84) of non-Rangers. Sixty percent (n = 26) of Rangers reported high family wellbeing compared to 44% (n = 70) of non-Rangers (Table 3).

### 3.4. Associations between Ranger Status and Wellbeing

#### 3.4.1. Life Satisfaction

**Figure 1:**
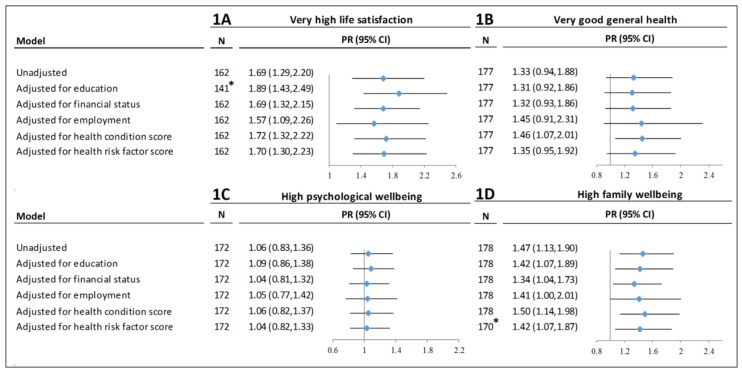
Associations between Ranger status and wellbeing measures. *PR = Prevalence Ratio.* * Missing category for relevant exposure variable was excluded due to zero cells.

The prevalence of very high life satisfaction was significantly higher for Rangers compared to non-Rangers (Figure 1A). The unadjusted prevalence ratio for very high life satisfaction was 1.69 (95% CI: 1.29, 2.20) for Rangers compared to non-Rangers. The association remained significant after individual adjustment for education (PR = 1.89, 95% CI: 1.43, 2.49), financial status (PR = 1.69, 95% CI: 1.32, 2.15), employment (PR = 1.57, 95% CI: 1.09, 2.26), health conditions score (PR = 1.72, 95% CI: 1.32, 2.22) and health risk factor score (PR = 1.70, 95% CI: 1.30, 2.23).

#### 3.4.2. General Health

There was not a significant association between Ranger status and general health in the unadjusted model (PR = 1.33, 95% CI: 0.94, 1.88), or in the models adjusted for education, financial status, employment, or health risk factor score (Figure 1B). However, after adjusting for health conditions, the prevalence of very good general health was higher for Rangers compared to non-Rangers (PR = 1.46, 95% CI: 1.07, 2.01).

#### 3.4.3. Psychological Wellbeing

There was not a significant difference in the prevalence of high psychological wellbeing between Rangers and non-Rangers in the unadjusted model (PR = 1.06, 95% CI: 0.83, 1.36), or in models individually adjusted for each exposure (Figure 1C).

#### 3.4.4. Family Wellbeing

The prevalence of high family wellbeing was significantly higher for Rangers compared to non-Rangers (Figure 1D). The unadjusted prevalence ratio for high family wellbeing was 1.47 (95% CI: 1.13, 1.90) for Rangers compared to non-Rangers. The association remained significant after individual adjustment for education (PR = 1.42, 95% CI: 1.07, 1.89), employment (PR = 1.41, 95% CI: 1.00, 2.01), financial status (PR = 1.34, 95% CI: 1.04, 1.73), health conditions score (PR = 1.50, 95% CI: 1.14, 1.98) and health risk factor score (PR = 1.42, 95% CI: 1.07, 1.87).

## 4. Discussion

We identified significant associations between Ranger participation and two wellbeing outcomes: very high life satisfaction, and high family wellbeing. In addition, the magnitude and direction of associations were similar for very good general health, but confidence intervals were wide, and results were not statistically significant for most models. While the analysis was not powered to adjust for multiple potential confounders simultaneously, associations between Ranger participation and health and wellbeing outcomes persisted after individual adjustment for key sociodemographic and health factors. Our findings are consistent with previous work and supports the argument that involvement in caring for country initiatives is associated with health and wellbeing [6,27,28]. Findings indicate that these health and wellbeing benefits may be independent of the employment and income benefits associated with participation in the Ranger program. The results were unsurprising to the Rangers, who have long argued and sensed the benefits of being involved in the Ranger program for themselves, their family and their community. However, this is the first time these associations have been quantified.

We did not identify a significant association between Ranger participation and high psychological wellbeing. This may be explained by external factors related or unrelated to Ranger work, poor question response, limitations of the measurement tool or other unknown factors. All represent areas for future enquiry and research.

It is possible that the different gender composition of the Ranger and non-Ranger samples could contribute to differences in wellbeing between the two groups. We tested this using the same analytical approach as we used to test if other key factors (education, employment status, financial status, health condition score, and health risk factor score) explained the difference between Rangers and non-Rangers in the four wellbeing outcomes. We found no material changes to the associations, indicating that differences between the two groups were not attributable to gender.

There was a high prevalence of health conditions and health risk factors among both Rangers and non-Rangers. The prevalence of heart disease was similar for Rangers compared to non-Rangers (≤11% vs. 11%), 28% of Rangers and 18% of non-Rangers reported diabetes, and 35% and 21% reported at least one of the two conditions, respectively. The prevalence of diabetes observed in the Ranger group exceeds prevalence estimates for Northern Territory residents (19%) in the 2012–13 National Aboriginal and Torres Strait Islander Health Survey [29,30]. Although the prevalence of heart disease appeared similar for Rangers compared to non-Rangers, many risk factors for heart disease were particularly common in the Ranger group (e.g., high cholesterol 23% vs. 12%; high blood pressure 26% vs. 15%). The prevalence of current smoking was high in both groups (63% among Ranger and 56% among non-Rangers), and consistent with estimates for Aboriginal and Torres Strait Islander adults (aged 15 years and above) in Alice Springs (54–64%) and Central Australia (43–47%) [28,29]. Adjusting for the cumulative measure of health conditions or health risk factors did not materially change the association between Ranger participation and wellbeing outcomes. This suggests that any differences in health conditions or health risk factors between Rangers and non-Rangers did not explain differences in wellbeing outcomes. The small sample in our study restricted any further analysis.

This ‘proof of concept’ study contributes to the evidence on the broader benefits of Ranger programs. Economic and biodiversity benefits of Ranger work have been well-established [10,11,12,13,14,15]. This ‘proof of concept’ study provides novel quantitative evidence on the potential health and wellbeing benefits of participation in a Ranger program. These findings add strength to ongoing assertions from community, Aboriginal organizations and conservation groups that the Australia Government’s Working on Country program is contributing towards closing gaps in health, employment and education [31,32]. Stability and expansion in policies that facilitate the development, implementation and sustainability of Ranger programs are likely to lead to improved wellbeing, health, and other gains for Aboriginal and Torres Strait Islander peoples.

While this analysis was based on a small sample, it provides support for the assertion that participation in the Ranger program is associated with improved wellbeing outcomes. We hypothesize that this is at least partly due to increased cultural engagement through the Ranger participation. It is likely there are many Aboriginal and Torres Strait Islander peoples engaged culturally; however, Ranger groups are routinely engaged in these activities. Ranger cultural engagement is facilitated by access to vehicles, to country and to those with cultural knowledge that enables them to perform their role. While not everyone can participate in Ranger programs, further investment in programs such as Ranger groups may enable wellbeing benefits among individuals and community. These benefits might come about from the promotion and transfer of cultural knowledge and skills or specific community-based activities such as school excursions to country, walking tours, guided by Rangers in partnership with traditional owners (cultural knowledge exchange). Further, if participation in the Ranger program impacts on health and wellbeing through increased cultural engagement, this would suggest that other forms of cultural engagement (outside of the Ranger program) may also be associated with benefits for health and wellbeing.

It should be noted that we were unable to determine the direction of association between Ranger participation and higher health and wellbeing outcomes due to the cross-sectional nature of the study. It is possible that Aboriginal and Torres Strait Islander people in better physical health (vs poorer) are more likely to become involved in the Ranger program, and as a result have better health and wellbeing outcomes. However, we found that Rangers had a similar, if not worse, health condition and health risk factor profile compared to non-Rangers. Further, we found that adjustment for health condition and health risk factor scores did not materially change the association between Ranger participation and health and wellbeing outcomes. Findings would be strengthened through testing for a dose-response relationship between time spent in the Ranger program and health and wellbeing outcomes, and through longitudinal analysis of cultural participation and health and wellbeing outcomes, as has been previously suggested [6]. While further evidence is required to demonstrate a causal association between Ranger participation and health benefits, taken together with previous evidence on economic and biodiversity benefits, it provides further support for the contribution of the Working on Country program to benefits beyond economic and biodiversity benefits [9].

## 5. Strengths and Limitations

A strength of the study is the participatory research approach and the ongoing community engagement and feedback. The research team ensured a two-way collaborative process with the Central Land Council and Rangers group in the establishment, implementation, and reporting of the research in conjunction with another community research organization. This value is reciprocal and joint seminars/presentations are planned to disseminate key messages on the research findings.

Indigenous field worker sampling was used for recruitment of non-Rangers. Indigenous field worker sampling uses formally trained field workers from the local community to identify ‘hard-to-reach’ populations. Indigenous field workers use local knowledge and networks to reach target populations [33]. Multiple sites are used for recruitment to enable a wide coverage of participants and therefore a larger sample population [34]. A purposive sampling approach was utilized to reach a quota of ~160 non-Ranger participants.

As a ‘proof of concept’ project, the research team were able to test the survey questions for the Mayi Kuwayu Study, and refine them for subsequent use in the upcoming national study [21], and potentially additional future research studies. The engagement and collaboration with Aboriginal and Torres Strait Islander communities and organizations has enabled the contextualization of research findings, and the generation of evidence that is meaningful and of value to participating communities.

A potential limitation of the study is the reliance on self-reported measures of health and wellbeing, using measures that have not all been robustly validated. However, these measures were conceptualized and developed through the conduct of over 20 focus groups with Aboriginal and Torres Strait Islander people across Australia, supporting the acceptance (face validity) of these measures [21]. Where possible, this study did employ measures of health and wellbeing that have been validated for use with Aboriginal and Torres Strait Islander adults (K5 scale), or that are commonly used (self-reported general health), with adaptations if requested through the consultation process. There is potential bias in the reporting of health conditions if participants had unequal access to healthcare/services.

This study was not intended to be representative of the entire Aboriginal and Torres Strait Islander population. While the prevalence of exposures and outcomes in this sample is not generalizable beyond the sample due to the lack of representativeness, representativeness is not necessary for reliable quantification of exposure-outcome relationships [35,36]. This study was focused on internal comparisons (i.e., differences between Rangers and non-Rangers); the results of these internal comparisons may be generalizable to other Aboriginal and Torres Strait Islander Ranger groups in Australia. This study was also not intended to provide evidence of causality or direction of association between Ranger participation and health and wellbeing. This can be explored in further research.

The relatively small sample size limited the statistical analysis that could be performed. While less than 5% data was missing for most variables, one exposure variable and one outcome variable had ≥20% missing data (financial status for Rangers, life satisfaction for Rangers and non-Rangers). However, the aim of this study was to provide ‘proof of concept’ on an association between participation in the Ranger program and health and wellbeing. Now that a ‘proof of concept’ association has been established, potential causal pathways can be explored in further detail in a larger sample [6,21].

## 6. Conclusions

This study identified significant associations between Ranger participation (compared to non-Rangers) in Central Australia and two wellbeing outcomes: very high life satisfaction, and high family wellbeing. We hypothesize that this association is at least in part explained by increased cultural engagement and expression through Ranger participation. In combination with previous evidence on economic and biodiversity evidence, this study contributes evidence on the multiple positive impacts of the Working on Country program on Aboriginal and Torres Strait Islander wellbeing.

As a ‘proof of concept’ study, this study was not intended to provide evidence on causality or mechanisms underlying an association between Ranger participation and health and wellbeing. This can be undertaken through further investigation on the role of cultural participation and expression on health and wellbeing in a larger sample of Aboriginal and Torres Strait Islander peoples. Longitudinal data is required to provide insight into causal relationships between cultural engagement, expression and wellbeing benefits.

## Figures and Tables

**Table 1:** Sociodemographic factors, by Ranger status.

Sociodemographic Factors	Rangers (n = 43)		Non-Rangers (n = 160)	
	%	n	%	n
Gender *				
Male	60	26	31	50
Female	28	12	65	104
Missing	12	5	4	6
**Age group (years)**				
16–24	≤12	≤5	17	27
25–34	33	14	29	47
35–44	16	7	17	27
>45	30	13	26	42
Missing	≤12	≤5	11	17
**Education**				
Not completed Year 12	44	19	54	87
Completed Year 12 or above	44	19	31	49
Missing	12	5	15	24
**Employment**				
Not employed	0	0	59	95
Employed	100	43	33	52
Missing	0	0	8	13
**Financial status**				
Low	37	16	54	86
High	42	18	31	49
Missing	21	9	16	25

* No substantial differences in PR and CI when gender was considered in outcome models.

**Table 2:** Health conditions and health risk factors, by Ranger status.

Health Conditions	Rangers (n = 43)		Non-Rangers (n = 160)	
	%	n	%	n
**Heart disease**				
No	84	36	84	134
Yes	≤12	≤5	11	17
Missing	≤12	≤5	6	9
**Diabetes**				
No	65	28	78	125
Yes	28	12	18	29
Missing	7	3	4	6
**Health condition score**				
No conditions	58	25	71	113
One or more health conditions	35	15	21	34
Missing	7	3	8	13
**High blood pressure**				
No	67	29	80	128
Yes	26	11	15	24
Missing	7	3	5	8
**High cholesterol**				
No	70	30	81	130
Yes	23	10	12	19
Missing	7	3	7	11
**Smoking status**				
Never smoked	16	7	36	58
Ex-smoker	≤12	≤5	7	11
Current smoker	63	27	56	89
Missing	≤12	≤5	1	2
**Health risk factor score**				
No health risk factors	14	6	29	46
One or more health risk factors	72	31	64	102
Missing	14	6	8	12

**Table 3:** Self-reported wellbeing, by Ranger status.

Self-Reported Wellbeing	Rangers (N = 43)		Non-Rangers (N = 160)	
	%	n	%	n
**Life satisfaction**				
Very high	60	26	36	58
Low to high	19	8	44	70
Missing	21	9	20	32
**General health**				
Very good general health	47	20	38	61
Poor to fair general health	35	15	51	81
Missing	19	8	11	18
Psychological wellbeing *				
High	63	27	53	84
Low to moderate	30	13	30	48
Missing	7	3	18	28
**Family wellbeing**				
High	60	26	44	70
Low to moderate	23	10	45	72
Missing	16	7	11	18

* High psychological wellbeing is classified as those who scored low to moderate psychological distress on the Kessler 5 scale.
